# Inflammable Gas Mixture Detection with a Single Catalytic Sensor Based on the Electric Field Effect

**DOI:** 10.3390/s140406409

**Published:** 2014-04-08

**Authors:** Ziyuan Tong, Min-Ming Tong, Wen Meng, Meng Li

**Affiliations:** 1 Engineering Institute of Information and Electricity, China University of Mining Technology, Xuzhou 221116, China; E-Mails: zton3078@uni.sydney.edu.au (Z.T.); ZS11060169@cumt.edu.cn (W.M.); derekli@cumt.edu.cn (M.L.); 2 School of Electrical and Information Engineering, University of Sydney, Sydney 2009, Australia

**Keywords:** gas analysis, catalytic sensor, safety monitoring in mines

## Abstract

This paper introduces a new way to analyze mixtures of inflammable gases with a single catalytic sensor. The analysis technology was based on a new finding that an electric field on the catalytic sensor can change the output sensitivity of the sensor. The analysis of mixed inflammable gases results from processing the output signals obtained by adjusting the electric field parameter of the catalytic sensor. For the signal process, we designed a group of equations based on the heat balance of catalytic sensor expressing the relationship between the output signals and the concentration of gases. With these equations and the outputs of different electric fields, the gas concentration in a mixture could be calculated. In experiments, a mixture of methane, butane and ethane was analyzed by this new method, and the results showed that the concentration of each gas in the mixture could be detected with a single catalytic sensor, and the maximum relative error was less than 5%.

## 1. Introduction

The analysis of mixtures of inflammable gases is very important for safety monitoring in mines because coal mine explosions are often caused by combustible gases. Therefore, gas monitoring systems must be installed in coal mines for the real-time monitoring of combustible gases [1]. There are usually three kinds of sensors used for gas monitoring in coal mines: infrared sensors, thermal conductivity sensors and catalytic sensors. The principle of the infrared gas sensor is to detect the infrared intensity after measuring the infrared spectrum of the gases. The principle of the thermal conductivity sensor is to detect the resistance variation due to the heat conduction of different gas concentrations. A catalytic sensor is a chemical sensor, which detects combustible gases by the resistance changes caused by the oxidation reaction of gases on the sensor. Infrared gas sensors are expensive, and their accuracy is easily influenced by the humidity in mines [2]. Thermal conductivity gas sensors are easily affected by the environment, and the ventilation and humidity in the mine could affect the reliability of the sensor [3]. With a simple structure, low cost and stable performance in mine environments, catalytic gas sensors are widely used for gas monitoring in mines.

The main cause for explosions in coal mines is the ignition of methane (CH_4_) so catalytic sensors are usually used to monitor methane [4]. However, coal residues often emit methane, C_2_H_6_, C_4_H_10_ and other combustible gases under high temperature [5]. The lower explosive limits of CH_4_, C_4_H_10_ and C_2_H_6_ are 5%, 3% and 1.8%, respectively, so the explosive concentrations of C_2_H_6_ or C_4_H_10_ are far lower than those of methane. Some research has shown that if CH_4_ was mixed with C_2_H_6_ or C_4_H_10_, the lower explosive limit of the gas mixture would be reduced and the risk of explosion would increase [6]. Therefore, monitoring methane with catalytic sensors for judging the possibility of explosions in mines will have larger errors. In order to solve this problem, it is necessary to analyze the concentration of each combustible gas in a gas mixture, then the explosive value will be obtained by the calculation of the concentration of each combustible gas, and the danger of explosion will be reasonably judged on the basis of the calculation result.

Although there are a variety of instruments for gas analysis on the market, they are not suitable for use in coal mines. At present, there is some research on the analysis of mixed gases in coal mines. Liu introduced a method for gas analysis based on infrared absorption technology [7]. The analysis system was made up of a broadband mid-infrared light source and a detector with a narrow-band filter. Each signal of the detector's outputs corresponded to a kind of gas according to its absorption wavelength. The gases were analyzed by a BP neural network using experimental data. The result showed that the relative error for gas analysis was less than 5%. Huang proposed a new method for gas analysis based on support vector machine (SVM) and multi-sensor data fusion. He used a multi-class classifier to fuse data of a sensor array composed of gas sensors, a temperature sensor and humidity sensor. The data fusion effectively eliminated the influence of ambient temperature and humidity on the gas sensors, and resulted in the identification of the gases [9]. Tong introduced a new method for the analysis of mixed inflammable gases with a catalytic sensor [9]. The catalytic sensor worked at different temperatures and had different outputs. The sensor's outputs were processed with a BP artificial neural network and the gases were analyzed after the model and algorithm were established through data training. The experimental results with three mixed gases showed that the method could accurately identify and analyze mixed gases.

In the above research, the gas analysis with an infrared sensor or thermal conductivity sensor could be influenced by the humidity and is not suitable for application in coal mines. The method using a catalytic sensor at different working temperatures seems to be feasible for the analysis of mixed combustible gases, but before any catalytic sensor can be used, it must undergo an aging process in which the sensor works for one week at a rated temperature of 400 °C and reaches a steady state, as changing its working temperature will make the output instable and influence its normal work [10].

**Figure 1.**
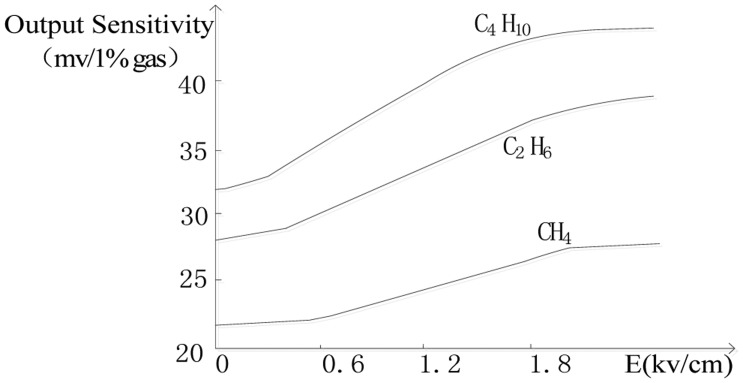
Electric field effects on three different gases.

In our study of catalytic sensors, we found that different electric fields could change the rate of the oxidation reaction of combustible gases on the sensor's surface and affect the output sensitivity of the catalytic sensor [11]. Figure 1 shows the output of a Wheatstone bridge circuit with a catalytic sensor exposed to CH_4_, C_4_H_10_ and C_2_H_6_ under different electric fields. The research results showed that as the field strength increased, the output sensitivity of the catalytic sensor for combustible gases increased. We will use these findings for the analysis of mixed combustible gases

## 2. Operating Principle of the Catalytic Sensor

**Figure 2.**
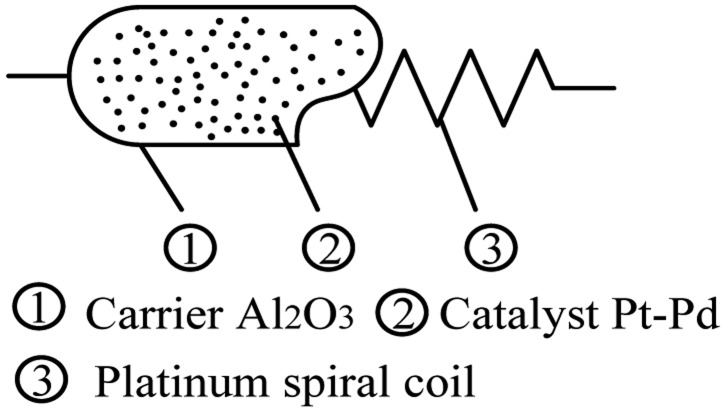
Structure of a catalytic sensor.

The catalytic sensor is obtained by coating Al_2_O_3_ as carrier on a platinum wire and depositing Pt or Pd in the catalyst, as shown in Figure 2.

Its working principle is an oxidation reaction of the flammable gases on the sensor under the action of the sensor's catalyst and high temperature caused by the electrical current on the sensor, which is about 400 °C [12]. The oxidation reaction of combustible gases will produce a lot of heat to increase the sensor's temperature, and then the resistance of the sensor will increase with the temperature characteristics of the platinum in the sensor. The concentration of combustible gas could be detected when the change sensor's resistance is converted into electrical signals with some kind of circuit.

**Figure 3.**
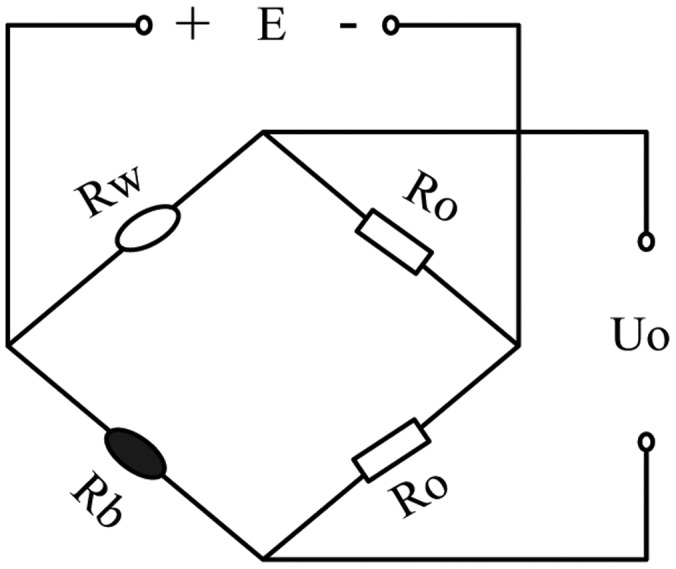
Wheatstone bridge with catalytic element.

The detection circuits for a catalytic sensor are usually of two kinds. The first kind is a Wheatstone bridge circuit and the other is a constant temperature detection circuit with automatic regulation of the sensor's current. Wheatstone bridge detection circuit as shown in Figure 3, which is made up of a compensating element (*R*_w_), catalytic sensor (*R*_b_) and two resistances *R*_o_ [13]. The compensating element is made by coating Al_2_O_3_ as carrier on a platinum wire and can eliminate the influence of environmental temperature on the catalytic sensor.

The bridge circuit is used to detect methane as an example. The catalytic sensor is electrically heated to about 400 °C, and at this temperature catalytic oxidation of the methane occurs readily. The oxidation reaction is as seen in Equation (1). The oxidation reaction of methane causes the increase of the temperature and resistance of the sensor, which is then measured by incorporating the element in the Wheatstone bridge network where the potential difference across the bridge forms the output of the device:

(1) $$CH_{4}+O_{2}\overset{Pt-Pd}{⟶}2H_{2}O+CO_{2}+795.5kJ$$

The resistance of the sensor increases to *R*_b_ + △*R*_b_, but the remaining resistances in the bridge remain the same, thus the bridge becomes unbalanced and the output voltage can be described as:

(2) $$U_{0}=\frac{R_{b}+\Delta R_{b}}{R_{w}+(R_{b}+\Delta R_{b})}E-\frac{E}{2}$$

In this way, the methane concentration can be detected by measuring the variation of output voltage from this unbalanced Wheatstone bridge.

**Figure 4.**
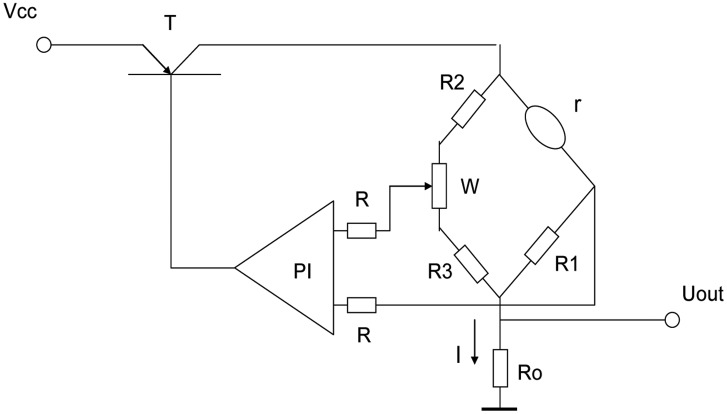
Constant temperature detection circuit.

The constant temperature detection circuit is shown in Figure 4. The detection circuit is composed of a bridge, which is formed with resistors *R*_1_, *R*_2_, *R*_3_, potentiometer W and catalytic sensor, PI regulator, regulating switch T and the sampling resistance *R*_0_. According to the 400 °C working temperature current of the catalytic element sensor, we take the current flowing through the catalytic element sensor as the initial current (*I* = *I*_0_) by adjusting the potentiometer W. Assume *R*_2_ = *R*_3_ ≫ *R*_1_ = *r*, then the current through the catalytic element sensor is approximately equal to that going through the sampling resistor *R*_0_ [14].

When in the detection of the combustible gas the gas starts to oxidize on the catalytic element sensor it will tend to generate heat which will increase the catalytic element's temperature and the resistance of the sensor, but by controlling and adjusting switch T and the PI regulator, then the working current through the catalytic element sensor will automatically reduce to maintain its temperature and resistance constant. The reduced current *I* reflects the concentration of measured gas, so the output of the constant temperature detection circuit is *U*_out_ = *IR*_0_.

In the above two detection circuits, the constant temperature detection circuit is better than the Wheatstone bridge circuit. In the Wheatstone bridge circuit, the temperature of the catalytic sensor will increase with the increase of concentration of combustible gas, and thermal stability and activity of the catalytic elements decline. In the constant temperature detection circuit, the temperature of the catalytic sensor is stable and its stability is much improved, so we will use the constant temperature detection circuit for the analysis of mixed combustible gases.

## 3. Gas Detection Principle

The static thermal equilibrium equation of catalytic sensor is shown below [15]:

(3) $$I^{2}r+Q=\alpha S(T-T_{0})+A\sigma S(T^{4}-T_{0}^{4})$$

The left side of Equation (3) is the total heat energy, which is the sum of the heat *I*^2^*r* generated by working current through the sensor and the energy *Q* produced by the oxidation reaction of the combustible gas on the catalytic sensor's surface. The right side is the total heat loss of the conduction heat loss and the radiation heat loss of the sensor. *I* is the current through the sensor, *r* is the resistance of the catalytic sensor, *T* is the temperature of catalytic sensor, *T*_0_ is the environment temperature, and *α*, *S*, *A*, *σ* are the physical parameters associated with the material structure of the catalytic sensor:

(4) Q=k(E)C

We set *k*(*E*) as the heat sensitivity coefficient which is associated with the electric field intensity and can be obtained through experiments, *C* as the measured gas concentration. $D=\alpha S(T-T_{0})=A\sigma S(T^{4}-T_{0}^{4})$; if the environment temperature is certain, *D* is a constant.

When analyzing the gas mixture with n kinds of mixed gases, we can get outputs *I*_1_ to *I*_n_ by adjusting the electric field n times. Then there are independent thermal equilibrium equations as follows:

(5) $$\begin{array}{c} \text{E}-{\text{Field}\;\text{E}}_{1}:{I_{1}}^{2}r_{1}+k_{11}(E_{1})C_{1}+k_{12}(E_{1})C_{2}+\ldots \ldots k_{1n}(E_{1})C_{n}=D\\ \text{E}-{\text{Field}\;\text{E}}_{2}:{I_{2}}^{2}r_{2}+k_{21}(E_{2})C_{1}+k_{22}(E_{2})C_{2}+\ldots \ldots k_{2n}(E_{2})C_{n}=D\\ \ldots \ldots \\ \text{E}-{\text{Field}\;\text{E}}_{\text{n}}:{I_{n}}^{2}r_{n}+k_{n1}(E_{n})C_{1}+k_{n2}(E_{n})C_{2}+\ldots \ldots k_{nn}(E_{n2})C_{n}=D \end{array}$$

In Equation (5), the temperature is constant, so resistance *r*_i_ is unchanged. Then we can calculate the concentration *C*_i_ based on Cramer's Rule:

(6) $$A=\left|\begin{array}{ccc} k_{11}(E_{1}) & \cdots & k_{1n}(E_{1})\\ \vdots & & \vdots \\ k_{n1}(E_{n}) & \cdots & k_{nn}(E_{n}) \end{array}\right|$$

(7) $$A_{i}=\left|\begin{array}{ccccccc} k_{11}(E_{1}) & \cdots & k_{1i-1}(E_{1}) & D-I_{1}^{2}r_{1} & k_{1i+1}(E_{1}) & \cdots & k_{1n}(E_{1})\\ k_{21}(E_{2}) & \cdots & k_{2i-1}(E_{2}) & D-I_{2}^{2}r_{2} & k_{2i+1}(E_{2}) & \cdots & k_{2n}(E_{2})\\ \vdots & & \vdots & \vdots & \vdots & & \vdots \\ \vdots & & \vdots & \vdots & \vdots & & \vdots \\ k_{n1}(E_{n}) & \cdots & k_{ni-1}(E_{n}) & D-I_{n}^{2}r_{n} & k_{ni+1}(E_{n}) & \cdots & k_{nn}(E_{n}) \end{array}\right|(i=1,2\ldots n)$$

(8) $$C_{i}=A_{i}/A$$

As *I*_i_ = *U_out_*_(_*_i_*_)_/*R*_0_, *I*_i_ can be calculated from output voltage *U_out_* in Figure 4. The solution *C*_i_ is the concentration of each gas in mixture gases.

## 4. Experiment Results and Analysis

**Figure 5.**
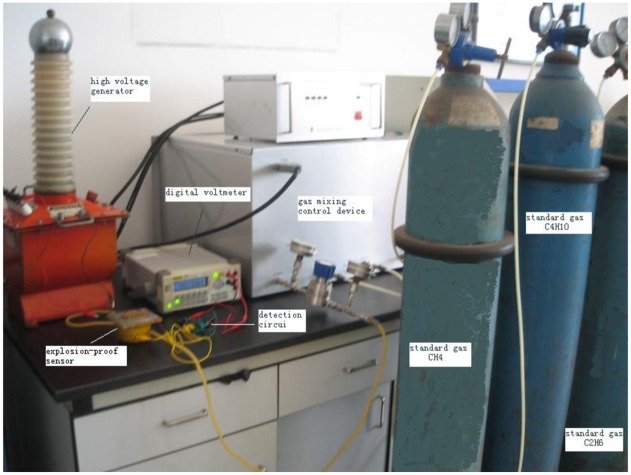
Experimental system for the gas analysis.

We take a mixture of gases containing CH_4_, C_4_H_10_ and C_2_H_6_ for the analysis experiments with a SH-3 catalytic sensor. The control voltages for the electrical field are 0 KV, 1.2 KV and 2.0 KV. The experimental system includes a high voltage generator, digital voltmeter, standard gases CH_4_, C_4_H_10_ and C_2_H_6_, a gas mixing control device, detection circuit and an explosion-proof sensor, as shown in Figure 5.

**Figure 6.**
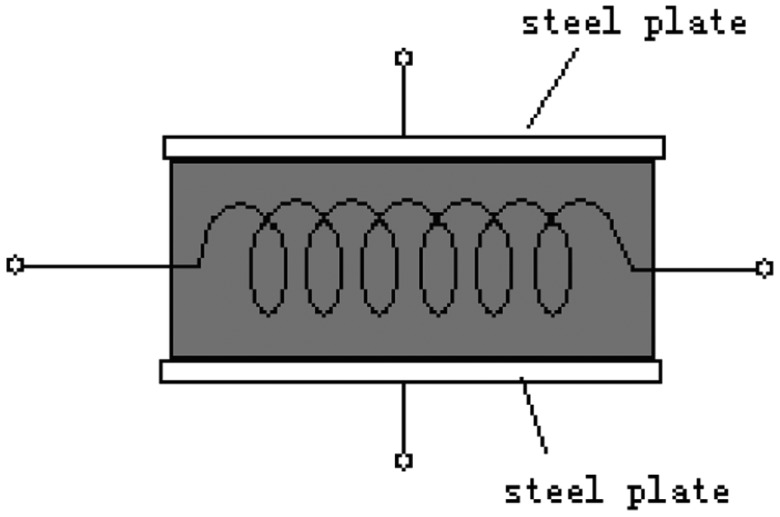
The structure of the special sensor.

To study the gases' analysis by the electric field effect on the catalytic sensor, we need a specific catalytic sensor. This sensor was made from a normal sensor with two 0.2 mm thick stainless steel plates on both sides, as shown in Figure 6. We can provide an electrical field on the sensor using the two plates.

First, we obtained the individual heat sensitivity coefficients *k_ij_* of CH_4_, C_4_H_10_ and C_2_H_6_ in different E-fields by the measurement of 1% concentration of each gas. The coefficients of methane *k*_11_, *k*_21_ and *k*_31_ correspond to 22 mv/1%, 24 mv/1%, 26 mv/1%; the coefficients of butane *k*_12_, *k*_22_ and *k*_32_ correspond to 32 mv/1%, 35 mv/1%, 38 mv/1%; the coefficients of ethane *k*_13_, *k*_23_ and *k*_33_ correspond to 28 mv/1%, 30 mv/1%, 32 mv/1%. Then we detected the mixture of gases by adjusting the electric field on the sensor and get the output signals *I*_1_, *I*_2_ and *I*_3_, Lastly we obtained the concentrations of methane, butane and ethane by solving Equation (3). The results are shown in Table 1.

From the results, we can see that there is an error because of the nonlinearity of the catalytic sensor, but the maximum relative error is less than 4% and this meets the requirements of relevant standards. The experiment showed that we could obtain the concentration of each gas in a mixture using the constant temperature detection circuit and the control of the electric field on the sensor. Catalytic sensors on the market usually have a compensation element whose role is to eliminate the influence of the environmental temperature on the catalytic sensor.

In the bridge detection circuit, a compensation element is used with the catalytic sensor. Its output is not affected by the environmental temperature, but in the constant temperature detection circuit, without the compensating element, the output will be influenced by the environmental temperature, because the catalytic sensor is made of platinum wire which is sensitive to temperature. How to solve this problem? Can the compensating element also be set into the bridge of the constant temperature detection circuit? Suppose *R*_1_ was replaced by the compensation element in Figure 4, then the bridge was balanced with *R*_1_ = *r* through the potentiometer *W*.

If such a circuit was used to detect combustible gas, the sensor's resistance would increase to *r* > *R*_1_, the bridge would have no balance, the circuit would automatically decrease the sensor's current and attempt to decrease *r* and restore the balance of the bridge. Because the platinum wires in the catalytic sensor and the compensating element are identical, the changes of their current would make their resistance undergo the same change, and finally the current would be adjusted to zero and the detection circuit would stop working. This is the reason why the compensating element was not used in the constant temperature detection circuit.

In order to solve the problem of temperature compensation of the temperature detection circuit, a platinum resistance with a great resistance was used to replace *R*_2_, also *R*_2_ = *R*_3_. In this circuit, if the environmental temperature changes, the change rate of resistance of the catalytic sensor is the same as *R*_2_. Assuming the same change rate is *α*, *αR*_1_*R*_2_ = *αrR*_3_, the bridge remains in balance with unchanged current that is the result of temperature compensation. The temperature compensation was proved by the experimental results in Table 2.

In the experiments in Table 2, the indoor temperature is 22 °C. The detection circuit was adjusted so the outputs were almost same whether the compensation resistor was used in the circuits or not, but when the detection circuit without compensation resistor was put into the constant temperature test box at the temperatures of 40 °C or 0 °C, the output error increase was more than the allowed error of relevant standards. After adding the compensation resistor, the output of the detection circuit was almost not influenced by the temperature, the maximum relative error was less than 5% and suitable for the practical requirements.

## 5. Conclusions

Based on our discovery that an electrical field could affect the sensibility of a catalytic sensor, we used a constant temperature detection circuit and provided an adjustable electric field to the sensor for the analysis of mixtures of gases. From the output signals of the detection circuit, the concentration of each gas in a mixture could be established by calculating the static thermal balance equation of the catalytic sensor. The results of experiments with methane, butane and ethane proved that an inflammable gas mixture could be analyzed with a single catalytic sensor. In order to solve the problem of the influence of environmental temperature on the catalytic sensor, a platinum resistance was used to replace the resistor and it showed good temperature compensation.

## Figures and Tables

**Table 1.** Analysis results of combustible mixtures.

**Actual Concentration /%**			**Test Results /%**			**Maximum Relative Error /%**
CH_4_	C_4_H_10_	C_2_H_6_	CH_4_	C_4_H_10_	C_2_H_6_	
1.10	0.85	1.85	1.14	0.82	1.83	3.63
2.00	0.64	1.58	1.95	0.62	1.54	3.52
2.80	0.50	1.05	2.72	0.46	1.02	2.85

**Table 2.** Analysis result of combustible mixtures under different temperatures.

			**Gas**			**Maximum Relative Error/%**

			**CH_4_**	**C_4_H_10_**	**C_2_H_6_**	
**Actual Concentration (%)**			1.10	0.85	1.85	n/a

**Temperature**	**0 °C**	**No Compensation**	1.01	0.78	1.75	8.24
		**Compensation**	1.13	0.82	1.81	3.53

	**22 °C**	**No Compensation**	1.14	0.82	1.83	3.64
		**Compensation**	1.14	0.82	1.83	3.64

	**40 °C**	**No Compensation**	1.20	0.91	2.02	9.19
		**Compensation**	1.15	0.83	1.84	4.55
